# A207 COMMON BILE DUCT OBSTRUCTION FROM RADIOGRAPHICALLY OCCULT LITHIASIS: A CASE REPORT AND CAUTIONARY TALE

**DOI:** 10.1093/jcag/gwab049.206

**Published:** 2022-02-21

**Authors:** D Wang, S Jalali, K Tsoi

**Affiliations:** Gastroenterology, McMaster University, Hamilton, ON, Canada

## Abstract

**Background:**

Biliary obstruction from choledocholithiasis (CDL) can lead to many serious complications, such as cholangitis and pancreatitis. Among noninvasive diagnostic tests for CDL, MRCP is superior to US and CT with a sensitivity of 89–95%. Despite this, MRCP may not always identify the cause of obstruction, leading to diagnostic dilemmas.

**Aims:**

We aim to review the various ways that CDL can hide on imaging, specifically MRCP.

**Methods:**

A 22-year-old male with pyruvate kinase deficiency was admitted to General Surgery for acute cholecystitis, for which he had a laparoscopic cholecystectomy. Preoperative MRCP showed a 3 mm common bile duct (CBD) with no stones; however, gross inspection of the gallbladder showed multiple green stones. Around one month later, he was readmitted to General Surgery for symptomatic biliary obstruction, with total bilirubin 923, ALP 457 and ALT 559. He was not febrile and did not require antibiotics. An MRCP showed a CBD of 10 mm with intra- and extrahepatic biliary dilation, but no obstructing focus was seen. Gastroenterology was consulted for undifferentiated hyperbilirubinemia. After further discussion, an ERCP with sphincterotomy was done the next day which also did not show obstruction. An abdominal US done the day after ERCP showed improvement in biliary dilation. After ERCP, the patient’s symptoms improved. Total bilirubin, ALP, and ALT one month later decreased to 121, 134, and 139 respectively. The hypothesis was that he had an obstructing gallstone that camouflaged within bile on imaging.

Consent was obtained from the patient to present this case report.

**Results:**

Although MRCP is one of the best noninvasive diagnostic tests for CDL, some stones may not appear on imaging. On MRCP, stones usually appear as hypointense spots surrounded by hyperintense bile on T2-weighted imaging. However, sludge can be isointense compared to bile. Additionally, stones impacted at the ampulla of Vater typically are not surrounded by bile and thus may evade detection on MRCP. Small stones <4 mm can be missed as well. CBD dilation greater than 10 mm is also associated with reduced sensitivity of MRCP for CDL. Even with ERCP, small stones may be missed unless seen on direct optical visualization after a blind balloon sweep.

Even if no obstructing lesion is seen on imaging, other clinical features can still suggest CDL requiring ERCP. The ASGE 2019 Guidelines on CDL recommend prompt ERCP for patients with total bilirubin >68.4 µmol/L and CBD dilation on imaging. Other radiographic signs suggesting CDL include CBD dilation, papillitis, and pericholecystic fat infiltration on CT, and MRCP findings of CDL-associated inflammation such as biliary wall thickening and periductal edema.

**Conclusions:**

Even if MRCP does not show obvious obstruction, ERCP should still be considered if the likelihood of CDL remains high.

**Funding Agencies:**

None

